# *Caenorhabditis elegans* employs innate and learned aversion in response to bacterial toxic metabolites tambjamine and violacein

**DOI:** 10.1038/srep29284

**Published:** 2016-07-07

**Authors:** Francesco Ballestriero, Jadranka Nappi, Giuseppina Zampi, Paolo Bazzicalupo, Elia Di Schiavi, Suhelen Egan

**Affiliations:** 1 School of Biological, Earth and Environmental Science and Centre for Marine Bio-Innovation, University of New South Wales, Australia; 2 Institute of Biosciences and BioResources, National Research Council, Naples, Italy; 3 Institute of Genetics and Biophysics, National Research Council, Naples, Italy

## Abstract

Bacteriovorus eukaryotes such as nematodes are one of the major natural predators of bacteria. In their defense bacteria have evolved a number of strategies to avoid predation, including the production of deterrent or toxic metabolites, however little is known regarding the response of predators towards such bacterial defenses. Here we use the nematode *C. elegans* as a model to study a predators’ behavioral response towards two toxic bacterial metabolites, tambjamine YP1 and violacein. We found that *C. elegans* displays an innate avoidance behavior towards tambjamine YP1, however requires previous exposure to violacein before learning to avoid this metabolite. The learned avoidance of violacein is specific, reversible, is mediated via the nematode olfactory apparatus (aversive olfactory learning) and is reduced in the absence of the neurotransmitter serotonin. These multiple strategies to evade bacterial toxic metabolites represent a valuable behavioral adaptation allowing bacteriovorus predators to distinguish between good and bad food sources, thus contributing to the understanding of microbial predator-prey interactions.

In the natural environment predation by protozoa and nematodes is a major cause of bacterial mortality^1^,^2^ and a driver of microbial community structure^3^,^4^. Thus evolution has favored bacteria that are capable of defending themselves from predation often via the production of chemical deterrents or toxins^5^,^6^,^7^. In return bacterial predators, including nematodes, are thought to have evolved sophisticated strategies to neutralize^8^ or to avoid these toxins^9^,^10^,^11^.

Nematodes are common in compost, soils and aquatic systems, and form the second main group of bacteriovorous organisms, after protozoa^6^. The nematode *Caenorhabditis elegans*, commonly found in decomposing fruit^12^ largely depends on its chemosensory system to detect and feed on bacteria in its natural environment and therefore represents an excellent model in which to study metazoan strategies involved in the avoidance of bacterial chemical defences^5^,^6^,^13^.

To date, at least two distinct mechanisms of avoiding toxic or harmful bacteria have been reported in *C. elegans*. The first relies on the ability of the nematode to detect repellent chemical cues produced by microorganisms, which triggers avoidance behavior on first encounter (hereafter designated as innate aversion)^14^. The second mechanism involves the ingestion of harmful (or pathogenic) bacteria by the nematode, which induces a process of associative learning, leading to the ability to avoid bacterial-related cues on subsequent encounters^15^. When the associative learning involves the detection of chemical cues by olfactory neurons, it is designated olfactory-dependent aversive learning (hereafter designated as aversive olfactory learning)^16^,^17^. In innate aversion a compound can be sensed as a repellent based on the evolutionary history of the nematode, while in contrast, aversive olfactory learning repellence is based on the life history of the individual animal^9^,^11^. In the present study the term repellent is used to describe the characteristic of a compound that induces aversive behavior in the nematode in either innate aversion or aversive learning.

In order to sense various repellents or attractants, *C. elegans* makes use of a well-defined chemosensory network, based on 16 pairs of sensory neurons, which penetrate the cuticle to expose their sensory cilia to the environment^18^,^19^. The cilia are subcellular structures of sensory neurons where chemoreceptors, involved in the detection of a range of attractants, repellents and pheromones, are expressed^20^.

In addition to the chemosensory organ, previous research has shown that pathogen-induced aversive olfactory learning requires the neurotransmitter serotonin^16^. The gene *tph-1* encodes for a tryptophan hydrolase required for the biosynthesis of serotonin^21^. Unlike wild type animals, *C. elegans* with mutations in *tph-1* do not learn to associate the physiological effect of pathogens or toxins with olfactory cues^16^.

We have previously reported on the toxicity of the bacterial secondary metabolites tambjamine YP1 and violacein towards *C. elegans*^22^,^23^. These two biologically active metabolites are produced by phylogenetically diverse bacterial strains thriving in a range of habitats such as terrestrial, marine, fresh water and glacier environments and include *Pseudoalteromonas tunicata* D2^7^,^24^, *Microbulbifer sp*.^7^, *Chromobacterium violaceum*^25^, *Collimonas sp.*^26^, *Duganella sp*.^27^, *Iodobacter fluviatile*^28^, *Streptomyces sp*.^29^ and *Janthinobacterium lividum*^30^. Tambjamine YP1 and violacein compounds have been shown to function as a chemical defense towards grazing predators^7^,^31^,^32^, however, the predator-related strategies involved in the avoidance of tambjamine YP1 and violacein remain to be elucidated.

The present study therefore aimed to analyze the behavioral strategies that the nematode *C. elegans* mounts to avoid the toxic bacterial metabolites tambjamine YP1 and violacein. By employing the *C. elegans, tph-1* and *osm-6* mutants (defective in the production of serotonin and sensory cilia, respectively) we further investigate whether serotonin and the cilia of the chemosensory neurons are necessary for aversive learning behavior against these bacterial secondary metabolites. Our study shows that the nematode uses different behavioral strategies to avoid these metabolites, whereby naïve worms avoid tambjamine yet they require prior exposure before learning to avoid violacein. This adverse learning observed for violacein occurs via the olfactory apparatus, analogous to avoidance of pathogenic bacteria^13^,^16^. These new findings highlight the importance of different behavioral responses of predatory organisms towards prey defenses metabolites, thus contributing to the understanding of the complex nature of microbial predator and prey interactions.

## Results and Discussion

In its natural habitat the bacteriovorous nematode *C. elegans* interacts with diverse microbial sources including both good quality and toxic food^33^,^34^. Recognizing and distinguishing between different quality food sources and potential pathogenic bacteria represents a valuable behavioral adaptation^5^,^9^,^11^. However, the behavioral strategies related to microbial predator-prey interactions remain poorly understood and studies have so far focussed only on a handful of bacterial species such as *Pseudomonas aeruginosa, Serratia marcescens*, *Bacillus thuringiensis, Vibrio cholerae* and *Microbacterium nematophilum,* chosen predominantly for their pathogenic characteristics^9^,^11^,^35^,^36^,^37^,^38^. Furthermore, the natural products produced by bacteria that induce aversion behavior (either innate or learned) are largely unknown, with only a few microbial metabolites successfully identified in *P. aeruginosa*^14^,^17^ and *S. marcescens*^13^. Therefore the current study investigated the behavioral response of *C. elegans* towards two naturally occurring bacterial antinematode compounds, namely tambjamine YP1 and violacein^22^,^23^,^39^.

### *C. elegans* avoids the toxic tambjamine YP1 via an innate aversion response

**Figure 1:**
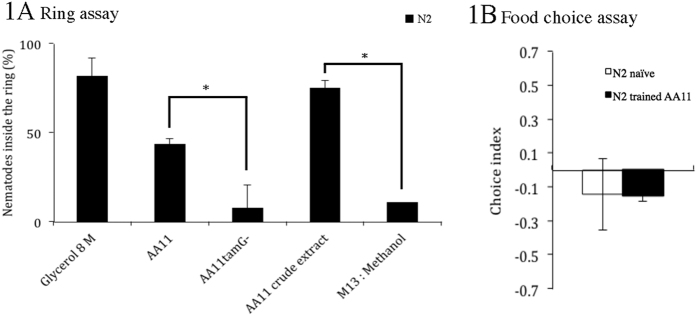
**(A)***C. elegans* assessed for aversion behavior using tambjamine-producing clone AA11 and crude extracts in ring assay. The ability to induce avoidance behavior was observed for AA11 clone and AA11 crude extract while AA11*tamG*^*−*^ and M13: Methanol (1:1) did not induce avoidance in the nematode. Glycerol was used as positive control. Each data point represents means ± the standard error of three replicates. * denotes a 0.001 < *p* < 0.05. Statistical significance was calculated comparing single treatments. **(B)** *C. elegans* assessed for aversive olfactory learning behavior in the food choice assay using tambjamine producing clone AA11. A choice index of −1.0 represents complete preference for the control bacterium, a choice index of 1.0 represents complete preference for the test bacterium, and an index of zero represents an equal distribution. The choice indexes of nematodes trained with AA11 clone (black bars) and naïve nematodes grown on AA11*tamG*^*−*^(white bars) were similar (*p*-value = 0.959) and no aversive learning was observed. Each data point represents means ± the standard error of three replicates.

To test the response of *C. elegans* towards tambjamine YP1 the nematode was challenged in a “ring assay” with either the tambjamine YP1 expressing *E. coli* clone (AA11), a non-toxic *E. coli* mutant clone (AA11*tamG*^*−*^), a tambjamine YP1-containing methanol extract or methanol as a negative control. After ten minutes, there was a significant difference (*p* = 0.025, Fig. 1A) between the number of nematodes located inside the ring of AA11 cells (43%) and those inside the ring of the tambjamine YP1 deficient mutant AA11*tamG*^*−*^ (7%). Similarly there was a significantly (*p* = 0.008) higher proportion of nematodes localized inside the ring of a crude extract of AA11 (75%) compared to the negative control (10%). These results indicate that naïve *C. elegans* avoid both bacterial cells expressing tambjamine YP1 as well as crude extracts containing the repellent. The stronger avoidance behavior observed against the crude extract compared to the AA11 cells is likely to be due to the combined result of both repellent (tambjamine YP1) and attractant (*E. coli* cells food) cues from AA11 cells, while the cell extract mainly contains the repellent tambjamine YP1.

In order to determine if prior contact with tambjamine YP1 influences avoidance response of *C. elegans* towards tambjamine YP1 a food choice assay was performed (Supplementary Figure S1). Trained animals were raised in the presence of both AA11 and AA11*tamG*^*−*^ while naïve animals were grown on AA11*tamG*^*−*^only. When nematodes were moved to the test plates, both trained and naïve nematodes showed a preference for the negative control AA11*tamG*^*−*^ and similarly negative choice indexes (naïve CI = −0.14, trained CI = −0.15, *p* = 0.959, Fig. 1B). The learning index was 0.01 (i.e. close to 0) indicating that previous exposure to the AA11 clone or tambjamine YP1 containing extract does not influence nematode avoidance behavior.

During the evolutionary history of *C. elegans,* innate chemosensory preferences may have evolved, in part to avoid potentially noxious situations including, for example the presence of pathogenic or toxic bacteria^9^,^10^,^11^. Indeed, innate aversion in *C. elegans* can be triggered by bacterial strains that are pathogenic for the nematode and which it may have encountered during its evolutionary history. Such a innate aversion has been demonstrated towards the pathogens *Bacillus thuringiensis*^35^ and *Microbacterium nematophilum*^36^.

Tambjamine and tambjamine-like compounds are widespread in the environment, where they have previously been shown to have antipredator activity^32^ including toxicity towards nematodes^22^. The results presented here indicate that at least in the case of tambjamine YP1, nematodes have evolved an innate avoidance response to this antipredatory compound. Interestingly the soil pathogen *S. marcescens* produces prodigiosin, a structural analogue of the tambjamine YP1 and recent studies have shown it to be effective against plant-parasitic nematodes^40^. Whilst further studies are required to elucidate if prodigiosin induces innate aversion in the nematode, it is plausible that this class of compounds has evolved as an informative ecological cue for bacterial predators such as *C. elegans,* enabling them to avoid potentially harmful food sources on their first encounter.

### *C. elegans* response to violacein is based on an aversive olfactory learning behavior that is reversible and uses the neurotransmitter serotonin

**Figure 2:**
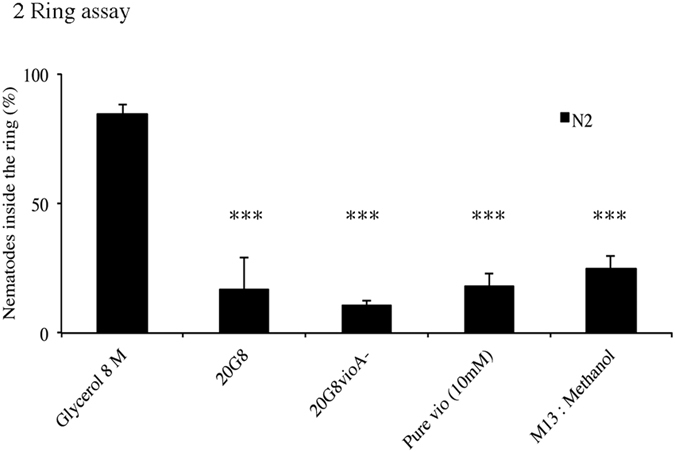
**(A)** Aversion behavior using violacein producing clone 20G8 and pure violacein. Innate aversion was not observed in nematodes exposed to either 20G8 clone, 20G8*vioA*^−^, or pure violacein 10mM. Each data point represents means ± the standard error of three replicates. ***denotes a *p* < 0.001 compared to Glycerol used as a positive control. Statistical significance was calculated using a one-way ANOVA with a Tukeys HSD post hoc test.

We assessed the response of *C. elegans* towards a second anti-predatory bacterial metabolite - violacein. Nematodes were challenged in a “ring assay” with either a violacein expressing *E. coli* clone (20G8) or the non-toxic *E. coli* mutant clone (20G8*vioA*^−^). Very few nematodes were found inside a ring of clone 20G8 (7%) or pure violacein 10 mM (17%) after 10 minutes while 85% of nematodes remained within the positive control glycerol (ANOVA *p* < 0.001, Fig. 2), indicating that in contrast to the innate aversion response towards tambjamine YP1, on first contact *C. elegans* does not perceive violacein as a repellent.

**Figure 3:**
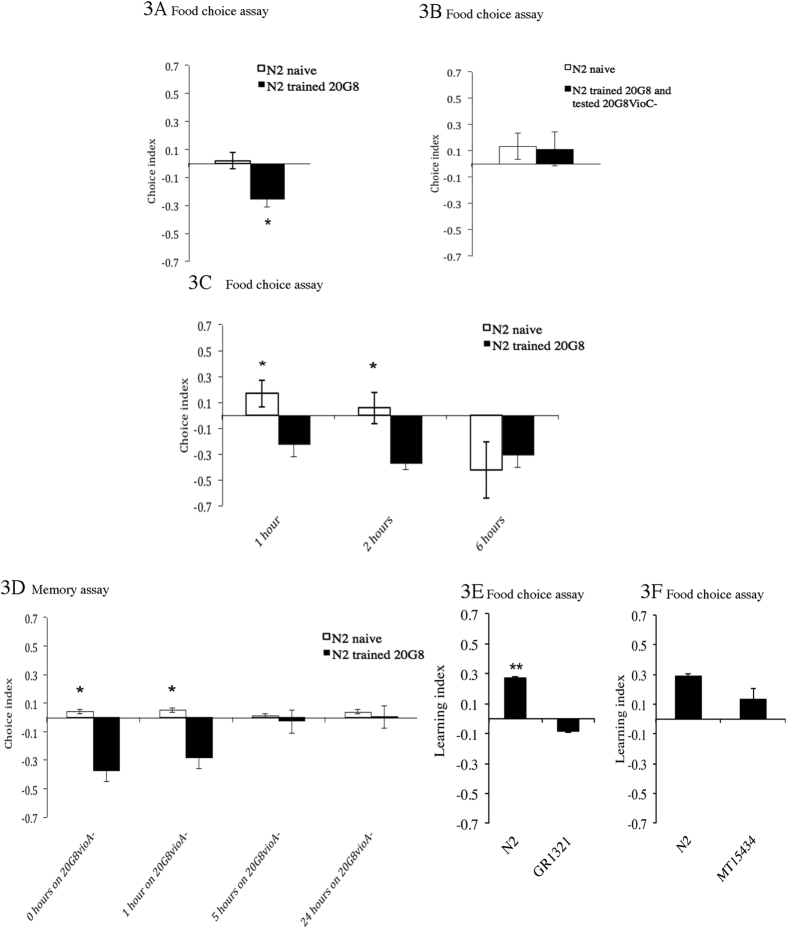
**(A)** Aversive olfactory learning in the food choice assay using violacein producing clone 20G8 (test) plus 20G8*vioA*^−^ (control). A choice index of −1.0 represents preference for the control bacterium, a choice index of 1.0 represents preference for the test bacterium, and an index of zero represents equal distribution. Animals trained with 20G8 (black bars) in the food choice assay avoid 20G8 more than the animals exposed to 20G8*vioA*^−^ (white bar) do. **(B)** *C. elegans* trained using 20G8 (black bar) and 20G8*vioA*^−^ (white bar) assessed for aversive olfactory learning behavior in the food choice assay tested using 20G8*vioC*^−^ (test) plus 20G8*vioA*^−^ (control). Nematodes grown in presence of 20G8 do not learn to avoid 20G8*vioC*^−^. **(C)** Food preferences assessed at different time points in the food choice assay using 20G8 (test) plus 20G8*vioA*^−^ (control). In order to keep track of the movements of nematodes during time anaesthetic was not used. At all time points nematodes trained using 20G8 (black bars) preferred the control bacteria over the test bacteria (CI always negative). Naïve nematodes, grown on 20G8*vioA*^−^ (white bars) changed their preferences from the test bacteria at 1 hr (CI = 0.15) to the control bacteria at 6 hrs (CI = −0.4). **(D)** Aversive olfactory learning behavior in the memory assay. Nematodes were trained with 20G8 (black bars) or 20G8*vioA*^−^ (white bars), moved to control bacteria for different lengths of time and then the nematode preference for 20G8 or 20G8*vioA*^−^ was tested using 20G8 (test) plus 20G8*vioA*^−^ (control). N2 wild type and *tph-1* mutants GR1321 **(E)** and MT15434 **(F)** assessed for aversive learning in the food choice assay tested using 20G8 (test bacterial spot) plus 20G8*vioA*^−^ (control bacterial spot). The two serotonin mutants, GR1321 and MT15434 displayed a lower learning index compared to N2 animals (*p* = 0.05 and *p* = 0.09 respectively). For panels A, B, C, and D each data point represents means ± the standard error of three replicates. In all panels each data point represents means ± the standard error of three replicates. * denotes a 0.001 < *p* < 0.05.

In order to investigate whether violacein can trigger aversive olfactory learning in the nematode (i.e. *C. elegans* can learn to avoid violacein), a food choice assay was performed. Naïve nematodes displayed a choice index close to zero indicating equal distribution between control (20G8*vioA*^−^) and test (20G8) bacteria. In contrast nematodes that had previously experienced 20G8, preferred 20G8*vioA*^−^ (significant difference when naïve CI = 0.02 and trained animals CI = −0.25 are compared, *p* = 0.021, LI = 0.27, Fig. 3A). Moreover addition of exogenous violacein to 20G8*vioA*^−^ was sufficient to induce avoidance in 20G8-trained animals (Naïve CI = 0.07, trained CI = −0.37, *p* = 0.038, LI = 0.44 Supplementary Figure S2).

Interestingly the aversive olfactory learning for violacein appears to be specific, as worms trained to avoid violacein (i.e. clone 20G8) do not avoid clones expressing the non-toxic violacein intermediate proviolacein (i.e. clone 20G8*vioC*^*−*^) (20G8-trained CI = 0.11, naïve CI = 0.13, *p* = 0.679, LI = 0.02, Fig. 3B). This apparent specificity for violacein is similar to the aversive olfactory learning behaviors seen for serrawettin molecules produced by *S. marcescens*^13^ and in the detection of toxic *P. aeruginosa* strains^41^.

We sought to investigate how long it takes for nematodes to learn to recognize and leave violacein-containing toxic food. In order to study this, anaesthetic was not added to the colonies of the test plates, nematodes were free to move from one bacterial spot to the other and the position of nematodes was recorded at three time intervals. At one and two hours naïve nematodes were randomly distributed; however after six hours, nematodes were more abundant on 20G8*vioA*^−^ than on 20G8 indicating that naïve nematodes became trained and preferred 20G8*vioA*^−^ by that time (Fig. 3C) and that *C. elegans* takes between two and six hours to learn to avoid violacein. In contrast at all time points trained nematodes prefer 20G8*vioA*^−^ rather than 20G8 (Fig. 3C). This phenomenon remains consistent for at least two days, *i.e.* nematodes do not move back on 20G8 after they moved on 20G8*vioA*^−^ (and the CI of both naïve and trained nematodes does not return to a positive value).

In order to assess whether learning can be reversed, a memory assay was performed. After training, naïve (exposed to 20G8*vioA*^−^ alone) and trained (exposed to 20G8*vioA*^−^ and 20G8) animals were incubated on plates spread with 20G8*vioA*^−^ cells and, at three time points, they were moved and tested for their choice between 20G8 and 20G8*vioA*^−^ bacteria. After a period of five hours in the absence of the toxic clone (i.e. on 20G8*vioA*^−^ only) the learning index was close to 0 and there was no significant difference (*p* > 0.05) between originally naïve and trained nematodes (LI at 0 h = 0.41, LI at 1 h = 0.23, LI at 5 h = 0.03, Fig. 3D). This observation supports previous studies showing that aversive learning does not involve permanent changes. Rather aversive learning results in reversible changes to neural circuits that modulate behavior^16^,^42^,^43^ and can be reversed after a period of time. Such reversible plasticity has strong adaptive value as it allows animals to respond to rapidly changing environments such as those in which *C. elegans* thrives^44^.

It has been previously shown that serotonin signalling is involved in the aversive olfactory learning induced by *P. aeruginosa*^16^. Here the hypothesis that serotonin signalling is also involved in violacein aversive olfactory learning was studied using two mutants strains defective in the synthesis of the neurotransmitter. Both strains carry the same 1306 bp deletion in the gene coding for the serotonin biosynthetic enzyme TPH-1, while they have different genetic backgrounds. Training with 20G8 was performed on both *tph-1* mutant animals (to exclude that other linked mutations might modify the behavioral responses) and resulted in a lower learning index compared to N2 wild type strain (p = 0.05 for strain GR1321 and p = 0.09 for strain MT15434, Fig. 3E,F respectively). These findings suggest although one of these strains (i.e. MT15434) resulted in a positive learning index, serotonin production is nevertheless a requirement for optimal violacein aversive olfactory learning.

### Ciliated neurons are required for aversive olfactory learning towards violacein

**Figure 4:**
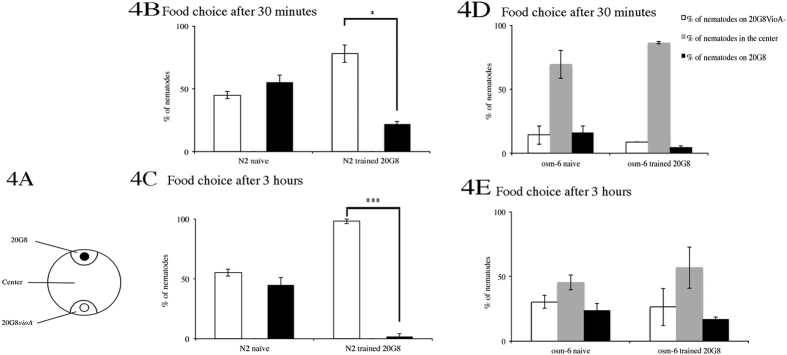
*C. elegans* wild type and mutant *osm-6* assessed for avoidance behavior in the food choice assay trained using 20G8-20G8*vioA*^−^ and tested using 20G8-20G8*vioA*^−^. After 20 minutes most of the *osm-6* nematodes remained in the centre of the test plate, therefore the results of this food choice were not represented as choice index. Instead the plate was divided in three sections **(A)** and the results were plotted as percentage of the nematodes’ distribution in the three sections after 30 minutes **(B**,**D)**, 3 hours **(C**,**E)**. No wild-type animals remained in the center of the plate at all times tested. Three replicates of each experiment were performed and statistical significance was calculated comparing single sections ***denotes a *p* < 0.0001.

Our results with wild type animals show that *C. elegans* can learn to avoid violacein. Aversive learning can be divided in two steps. In the first step the animal encounters the toxic compound and associates the noxious effect that the toxin produces with some cue such that in the second step it can recognize the cue and avoid the toxin before becoming affect. For *C. elegans*, in the cases documented so far^42^,^45^, these cues are olfactory in nature, detected by the ciliated olfactory neurons in a process known as aversive olfactory learning. To test whether ciliated olfactory neurons are also involved in the aversive olfactory learning towards violacein we tested the olfactory defective mutant *osm-6*^46^ in the food choice assay. After 30 minutes and 3 hours *osm-6* mutant animals randomly distribute on the two bacterial spots (*p* > 0.05 when nematodes numbers on 20G8 and 20G8*vioA*^−^ are compared, Fig. 4D,E), in contrast at the same time trained wild type animals prefer the negative control 20G8*vioA*^−^ (*p* < 0.05 when nematodes numbers on 20G8 and 20G8*vioA*^−^ are compared, Fig. 4B,C). In this experiment both wild type and *osm-6* trained animals have experienced the noxious effect of violacein during training (first step), however from the distance only the wild type animals are able to recognize and avoid it (second step) while the *osm-6* mutant nematodes lacking ciliated olfactory neurons are unable to detect it and despite their training background randomly distribute on the two bacterial spots. This result shows that the aversive olfactory learning towards violacein in wild type nematodes requires ciliated olfactory neurons. Our data with wild type nematodes is in line with previous studies showing that *C. elegans* learns to avoid pathogenic bacteria (e.g. *P. aeruginosa*)^42^ and poor quality food^10^ using the olfactory apparatus.

Evidence is accumulating that behavioral, cellular and immune responses maybe coordinated by common pathways, in particular by the insulin-like signalling (ISS) pathway^47^,^48^. Indeed we have recently documented that the cellular response of *C. elegans* towards violacein toxicity is, in part, mediated by the IIS pathway^23^. Thus it is possible that the behavioral responses to cues sensed by ciliated chemoreceptors such as those observed here are also modulated by the IIS pathway, however further studies will be required to confirm the specific involvement of IIS in the behavioral response of *C. elegans* against bacterial anti-predator metabolites such as violacein.

## Summary

Learning to associate sensory cues with threats is critical for minimizing aversive experience. Whilst the response of the animal model *C. elegans* towards pathogens has been extensively investigated, the behavioral response of this bacterial predator to defense metabolites originating from non-pathogenic, environmental bacteria has been largely overlooked. This study demonstrates that *C. elegans* employs multiple strategies to behaviorally avoid toxic bacterial defense metabolites. The bacterial metabolite tambjamine YP1 induces innate aversion in *C. elegans*. In contrast the nematode has no innate aversion towards violacein but can learn to avoid it by employing olfactory aversive learning, which is reversible, based on the olfactory neurons and can detect toxins at a distance. The use of multiple strategies by *C. elegans* to sense and respond to specific bacterial metabolites represent a successful adaptive strategy for bacterial predators in response to toxic prey metabolites.

## Methods

### Strains and culture conditions

Bacterial strains, *C. elegans* strains and vectors used in this study are listed in Supplementary Table S1. All strains were grown in Luria broth (LB10) or nematode growth medium (NGM)^49^ as indicated. Solid medium was prepared by the addition of 19 g/L (w/v) of agar (Oxoid, Australia). Where required, chloramphenicol (12.5 μg/mL), kanamycin (100 μg/mL), tetracycline (10 μg/mL) and L-arabinose (0.02% w/v) were added to the media. All bacterial strains were grown at 25 °C and stored in 30% (v/v) glycerol at −80 °C. *C. elegans* strains were maintained at 20 °C on NGM agar plates spread with *E. coli* OP50 as a food source^50^. *C. elegans* strains were stored in glycerol (70:30 v/v) at −80 °C.

Crude extracts containing tambjamine YP1 compound where prepared from *E. coli* clone AA11 cells as previously described^51^.

### Innate aversion - ring assay

The ring assay was adapted from the osmotic ring assay^15^ with minor modifications as described below. The ring assay was conducted on 9 cm petri dishes containing LB agar media. The crude extracts, pure compounds or bacterial cultures to be tested were re-suspended in M13 buffer or in a 1:1 ratio of M13 buffer and methanol. Plates were used after methanol had evaporated. Glycerol (8M) was employed as positive control^15^ and M13 buffer methanol diluent as the negative control for avoidance behavior. Three centimetre diameter rings of individual test crude extracts, pure compounds or bacterial cultures were drawn on the surface of the agar using the blunt end of a glass hockey stick. Plates with bacterial rings were grown for four days at 25 °C before use. Non-starved hermaphrodite animals (*i.e.* collected from agar plates where a bacterial lawn of *E. coli* OP50 cells remained clearly visible) at the stage of young adults were washed twice in S Basal. Thirty nematodes were added inside each ring and after ten minutes the position of each animal was recorded (either inside or outside the ring). Three replicates of each experiment were performed and statistical significance between treatments was calculated using the R software multiple comparison package^52^ with a one-way ANOVA followed by Tukeys HSD post hoc testing.

### Aversive olfactory learning - food choice assay

The food choice assay assesses if a compound or bacterial strain has induced aversive olfactory learning in the nematode and is based on previously described assays^16^, with the exception that, when required, bacterial colonies were used as odor sources. The food choice assay consists of two parts: (*Part one*) nematode training and (*Part two*) testing the nematode food choice (Supplementary Figure S1). Briefly,

#### Part one: nematode training

*C. elegans* grown on control bacteria and having never been exposed to the test bacteria are referred to as naïve nematodes. In contrast, trained *C. elegans* are grown in presence of both the control and the test bacteria. Control bacteria^16^ had to be included because violacein expressing *E. coli* alone do not support the growth of nematode to adulthood^23^. Unless otherwise indicated the control bacteria are either the non-toxic 20G8*vioA*^−^ or AA11*tamG*^*−*^ mutants (see Table S1).

Naïve *C. elegans* were grown from eggs according to Zhang *et al.*^16^ on a 3.5 cm LB10 agar plate of control bacteria (Supplementary Figure S1). Trained *C. elegans* were grown on LB10 agar plates spread half with 25 μL of an overnight liquid culture of the control bacteria and half with 25 μL of an overnight liquid culture of the test bacteria (Supplementary Figure S1). When animals reached adulthood they were transferred to test plates.

#### Part two: testing nematode food choice

In the second part of the food choice assay nematodes with different training backgrounds were allowed to choose between the control and test bacteria and their food choice was recorded. Specifically, *E. coli* control and test clones were grown separately overnight at 37 °C in LB10, 5 μL of liquid cultures were spotted on 3.5 cm LB10 agar plates and incubated for four days at 25 °C. When required, control bacteria and test bacteria were substituted with a solution of the test compound in methanol to a final volume of 5 μL. One drop of the control bacteria and one drop of the test bacteria or test compound per plate were spotted at a 2 cm distance at the exact opposite ends of the plate (Supplementary Figure S1). Plates with bacteria were grown for four days at 25 °C before use. Plates with solutions of test compounds in methanol where allowed to air dry 30 mins before use. Unless otherwise indicated, 1 μL of a 10 mM sodium azide (NaN_3_) anaesthetic solution was added to both the test and control bacterial spots to immobilize the nematodes once they reach either spots.

Adult nematodes were washed twice in S Basal medium and between 30 and 40 nematodes were placed on the plate exactly between the two test substances. Unless otherwise indicated, the number of nematodes on either the control or test spots was recorded after one hour. Both a choice index (CI) and learning index (LI) for each test was calculated as indicated below.

### Choice index and Learning index

CI is calculated as follows: CI = (number of nematodes at the test spot - number of nematodes at the control spot)/total number of nematodes in the assay^16^. A positive choice index indicates preference for the test bacteria/test chemical, a negative choice index indicates preference for the control bacteria/control chemical and an index of zero represents an equal distribution (Supplementary Figure S1)

Learning index (LI) is calculated as follow: LI = (choice index_naïve_ − choice index_trained_). A positive learning index indicates a learned avoidance of toxic bacteria with the value 2 as the maximum learning index^16^.

Each experiment was carried out in triplicate. Statistical significance between individual treatments and controls was calculated in Excel using a two-tailed Student’s *t*-test.

### Aversive olfactory learning-Memory assay

The memory assay was employed to test how long the aversive learning lasts in the nematode. In the memory assay the nematodes’ training was carried out as previously described for the food choice assay with the exception that after training the nematodes were moved to a plate of control bacteria for one, five and 24 hours before moving the nematodes to test plates.

## Additional Information

**How to cite this article**: Ballestriero, F. *et al.* *Caenorhabditis elegans* employs innate and learned aversion in response to bacterial toxic metabolites tambjamine and violacein. *Sci. Rep.* **6**, 29284; doi: 10.1038/srep29284 (2016).

## Supplementary Material

Supplementary Information
