# Supplementary material for: Caenorhabditis elegans employs innate and learned aversion in response to bacterial toxic metabolites tambjamine and violacein
Source: Sci Rep. 2016 Jul 7;6:29284. doi: 10.1038/srep29284 (PMC4935850; doi:10.1038/srep29284)
Supplement: Supplementary Information [file srep29284-s1.pdf]

Supplementary material

Title ***Caenorhabditis elegans*** **employs innate and learned aversion in response to bacterial toxic metabolites tambjamine and violacein**

Francesco Ballestriero[1], Jadranka Nappi[1], Giuseppina Zampi[3], Paolo Bazzicalupo[2,3], Elia Di Schiavi[2,3#] and Suhelen Egan[1#]

[1]School of Biological, Earth and Environmental Science and Centre for Marine Bio-Innovation, University of New South Wales, Australia, [2]Institute of Genetics and Biophysics, National Research Council, Naples, Italy, [3]Institute of Biosciences and BioResources, National Research Council, Naples, Italy

Running title: Nematode avoidance of bacterial toxic metabolites

[#] Corresponding authors email: s.egan@unsw.edu.au and elia.dischiavi@ibbr.cnr.it

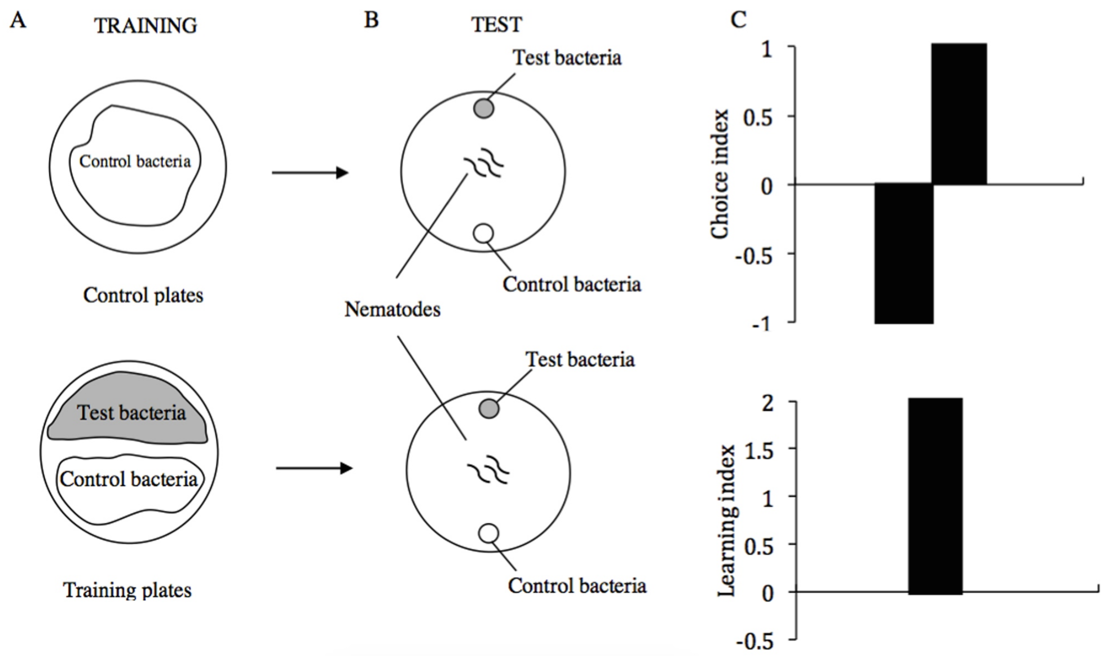


**Figure S1**. Schematic representation of the protocol for the food choice assay. (A) training plates, and (B) test plates. (C) Data from the food choice assay plotted as choice index or Learning index. A choice index of -1.0 represents complete preference for the control bacterium, an index of 1.0 represents complete preference for the test bacterium, and an index of zero represents an equal distribution. A positive learning index indicates a learned avoidance of toxic bacteria with the value 2 as maximum learning index. Adapted from (Zhang et al., 2005).

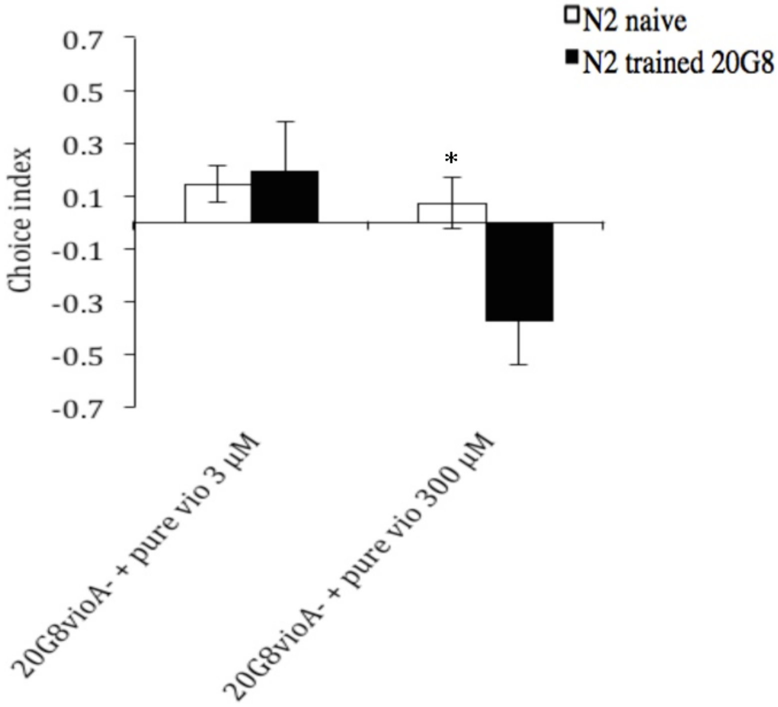


**Figure S2**. *C. elegans* assessed for aversive olfactory learning behavior in the food choice assay tested using 20G8vioA- and 20G8vioA- supplemented with pure violacein. Naïve (white bars) and trained (black bars). Nematode preference was tested against three and 300 μM of pure violacein. Each data point represents means ± the standard error of three replicates. * denotes $0.001<p<0.05$.

**Table S1.** Bacterial and nematode strains and vectors used in this study.

| Strain/Vector | Relevant characteristic or genotype | Source or reference |
|---|---|---|
| Bacterial strains | | |
| *E. coli EPI300-T1[R]* | F-*mcr*A Δ(*mrrhsd*RMS*mcr*BC) φ80d*lac*ZΔM15Δ*lac*X 74 *rec*A1 *end*A1 *ara*D139 Δ(*ara*, *leu*) 7697*gal*U *gal*K λ- *rps*L *nup*G *trf*A *ton*A *dhfr* | Epicentre |
| *E. coli* AA11 | Fosmid AA11 cloned in *EPI300-T1[R]*; Cm[r] | (Burke, et al. 2007) |
| *E. coli* AA11*tamG*[-] | Fosmid AA11 mutated in *tamG* gene and cloned in *EPI300-T1[R]*; Cm[r], Tet[r] | (Ballestriero, et al. 2014) |
| *E. coli* 20G8 | Fosmid 20G8 cloned in *EPI300-T1[R]*; Cm[r] | (Penesyan, et al. 2012) |
| *E. coli* 20G8*vioA*[-] | Fosmid 20G8 mutated in *vioA* gene and cloned in *EPI300-T1[R]*; Cm[r], Kan[r] | (Ballestriero, et al. 2014) |
| *E. coli* 20G8*vioC*[-] | Fosmid 20G8 mutated in *vioC* gene and cloned in *EPI300-T1[R]*; Cm[r], Kan[r] | (Ballestriero, et al. 2014) |
| *E. coli* OP50 | Uracil auxotroph | (Brenner 1974) |
| Nematode strains | | |
| N2 Bristol | *C. elegans* wild type isolate | CGC [b] |
| PR811 | *osm-6*(*p811*) V. *osm-6* mutants are defective in the ability to avoid high osmolarity. *osm-6* is required for proper sensory cilium structure. In *osm-6* mutants most of the ciliated neurons have severely shortened cilia that are not exposed to the environment. *osm-6* mutant shows reduced avoidance responses to repellents and fail to stain amphids with FITC. | CGC [b] |
| GR1321 | *tph-1*(*mg280*) *cam-1*(*vs166*) II. Mutation in *tph-1,* which encodes the enzyme tryptophan hydroxylase that catalyses the rate-limiting first step in serotonin biosynthesis. Some phenotypic defects originally attributed to *mg280* in this strain are likely due to *vs166*, a deletion in the *cam-1* gene. | CGC [b] |
| MT15434 | *tph-1*(*mg280*) II. Backcrossed strain carrying *mg280* allele without the *cam-1* mutation. | (Flavell, et al. 2013) [c] |
| Vectors | | |
| pCC1FOS[a] | Fosmid backbone for genomic library; Cm[r] | Epicentre |

[a] Copy number inducible by arabinose. [b] *Caenorhabditis* Genetics Center. [c] Strain provided by Cori Bargmann, Rockfeller University New York

## References

Ballestriero, F., Thomas, T., Burke, C., Egan, S. & Kjelleberg, S. Identification of compounds with bioactivity against the nematode *Caenorhabditis elegans* by a screen based on the functional genomics of the marine bacterium *Pseudoalteromonas tunicata* D2. *Appl. Environ. Microbiol.* **76**, 5710-5717 (2010).

Ballestriero, F. *et al.* Antinematode activity of violacein and the role of the insulin/IGF-1 pathway in controlling violacein sensitivity in *Caenorhabditis elegans*. *PLoS One* **9**, e109201 (2014).

Brenner, S. The genetics of *Caenorhabditis elegans*. *Genetics* **77**, 71-94 (1974).

Burke, C., Thomas, T., Egan, S. & Kjelleberg, S. The use of functional genomics for the identification of a gene cluster encoding for the biosynthesis of an antifungal tambjamine in the marine bacterium *Pseudoalteromonas tunicata*. *Environ. Microbiol.* **9**, 814-818 (2007).

Flavell, S. W., *et al* (2013). Serotonin and the neuropeptide PDF initiate and extend opposing behavioral states in *C. elegans*. *Cell* **154**: 1023-1035.

Penesyan, A. *et al.* Assessing the effectiveness of functional genetic screens for the identification of bioactive metabolites. *Mar Drugs.* **11**, 40-49 (2012).

Zhang, Y., Lu, H. & Bargmann, C. Pathogenic bacteria induce aversive olfactory learning in *Caenorhabditis elegans*. *Nature* **438**, 179-184 (2005).
